# ULBPs: regulators of human lymphocyte stress recognition

**DOI:** 10.18632/oncotarget.22417

**Published:** 2017-11-13

**Authors:** Jianmin Zuo, Benjamin E. Willcox, Paul Moss

**Affiliations:** Jianmin Zuo: Cancer Immunology and Immunotherapy Centre, Institute of Immunology and Immunotherapy, University of Birmingham, Edgbaston, Birmingham, UK

**Keywords:** NK, NKG2D, ULBPs, stress recognition, cancer immunotherapy

NKG2D is a critical activatory molecule expressed on cytotoxic cells such as NK cells, γδ T cells and subsets of αβ T cells [1]. Ligation of NKG2D acts to trigger cytotoxicity of NK cells and acts as a co-stimulatory molecule on T cells. The ligands that bind to NKG2D, known as NKG2DL, are grouped into two families and comprise eight different proteins termed MIC-A/B and ULBP1-6 (Figure 1a). Importantly, these proteins are expressed on the surface of cells in response to stress signals such as viral infection or transformation and act to trigger cellular elimination by cytotoxic effector cells. A striking feature of NKG2DL is the considerable polymorphism within both gene families [2] and specific alleles have been associated with increased risk of autoimmune disease or relapse after stem cell transplantation.

**Figure 1:**
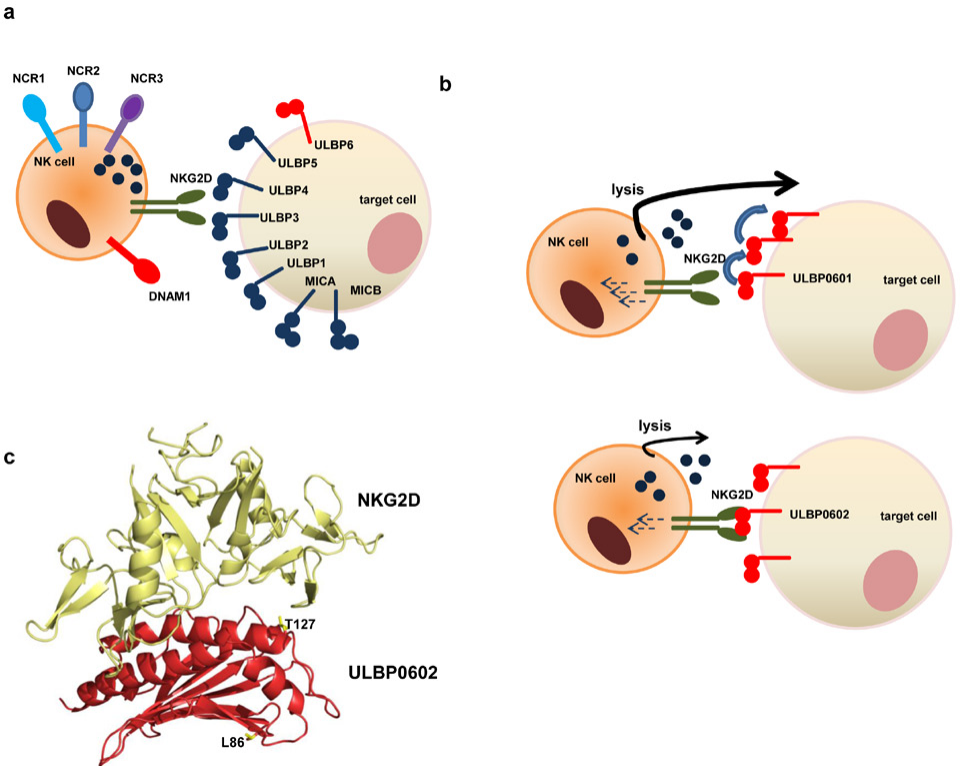
Summary of the functional importance of allelic variation within *ULBP6* **a.** The NKG2D ligand family consists of 8 members. **b.** The two major alleles of *ULBP6* encode the ULBP0601 and ULBP0602 proteins. ULBP0602 has a very high affinity interaction with the NKG2D receptor but paradoxically is associated with lower activation of the effector cytotoxic cell and reduced target cell lysis. **c.** Crystal structure of ULBP0602 showing the location of the two polymorphisms which create the energetic hotspot for ULBP6 binding.

ULBP6 is the most polymorphic gene within the ULBP family and in recent work our team contrasted the molecular and functional correlates of the two most common protein variants, ULBP601 and ULBP602 (Figure 1b) [3]. Strikingly, surface plasmon resonance and x-ray crystallography revealed that the R>L polymorphism within ULBP0602 generated an energetic hotspot within the NKG2D-ULBP6 interaction (Figure 1c), resulting in an affinity of 15.5 nM, between 10 and 1000-fold greater than the affinities of other ULBP-NKG2D interactions. Paradoxically, this very strong interaction between NKG2D and the ULBP0602 allele had the effect of impairing NKG2D-mediated effector functions (Figure 1b). This was most likely related to impaired serial engagement of ULBP6 binding, but conceivably cleavage and release of a high affinity soluble ULBP602 protein isoform from the cell surface capable of blocking NKG2D binding could also contribute. Although previous studies on MICA have highlighted that the hierarchy of responses to different polymorphic MICA variants varied between individuals [4], the enhanced response to ULBP0601 compared to ULBP0602 was universally observed across different donors [3], consistent with an important role in immune regulation.

The mechanisms that underlie the association of NKG2DL alleles with clinical disorders such as auto-immunity [5] or tumor-specific responses [6] are unclear but these data indicate that variation in the strength of the NKG2D-mediated cellular response is an important factor. This further points towards the potential development of novel therapeutic approaches including regulation of the expression level of NKG2DL proteins or modulation of the strength or extent of NKG2D-NKG2DL interaction.

The critical role of NKG2DL in tumour-specific immune responses is highlighted by the fact that tumours frequently evolve a range of mechanisms to evade NKG2D-mediated responses. These include NKG2DL cleavage from the surface, and NKG2D downregulation which is frequently observed on cytotoxic cells in patients with cancer [7]. Interestingly, recent *in vivo* murine studies have shown that ectopic expression of NKG2D ligands on tumor cells can be sufficient to mediate control of tumor growth[8]. In order to fully understand how the NKG2D-NKG2DL axis can be targeted in patients with cancer it will be necessary to increase understanding of the fundamental properties of these receptors. These studies will require a detailed profile of NKG2DL expression within different human tumours and how this may be influenced through procedures such as chemotherapy or radiotherapy.

The central importance of NKG2D interactions within immune homeostasis has been relatively under investigated but an emerging hope is that detailed interrogation of the significance of natural alleleic variation within the ULBPL family members [3] will serve to guide approaches to therapeutically manipulate NKG2D-NKG2DL interactions.

## References

[1] Raulet DH (2013). Annual Review of Immunology.

[2] Radosavljevic M (2002). Genomics.

[3] Zuo J (2017). Science Signaling.

[4] Shafi S (2011). Science Translational Medicine.

[5] Petukhova L (2010). Nature.

[6] Antoun A (2012). British Journal of Haematology.

[7] Parry HM (2016). Oncotarget.

[8] Cerwenka A (2001). Proceedings of the National Academy of Sciences USA.

